# Supplementary material for: Analysis of chi angle distributions in free amino acids via multiplet fitting of proton scalar couplings
Source: Magn Reson (Gott). 2024 Aug 19;5(2):103–20. doi: 10.5194/mr-5-103-2024 (PMC11570886; doi:10.5194/mr-5-103-2024)
Supplement: The supplement related to this article is available online at: https://doi.org/10.5194/mr-5-103-2024-supplement. [file mr-5-103-2024-supplement.zip › Syed2024/data/fit1d_fitnmr_output.tar.gz!/fit1d/leu/900/bmse000042_simulation_2_nmredata/1H/resonances_1d.pdf]

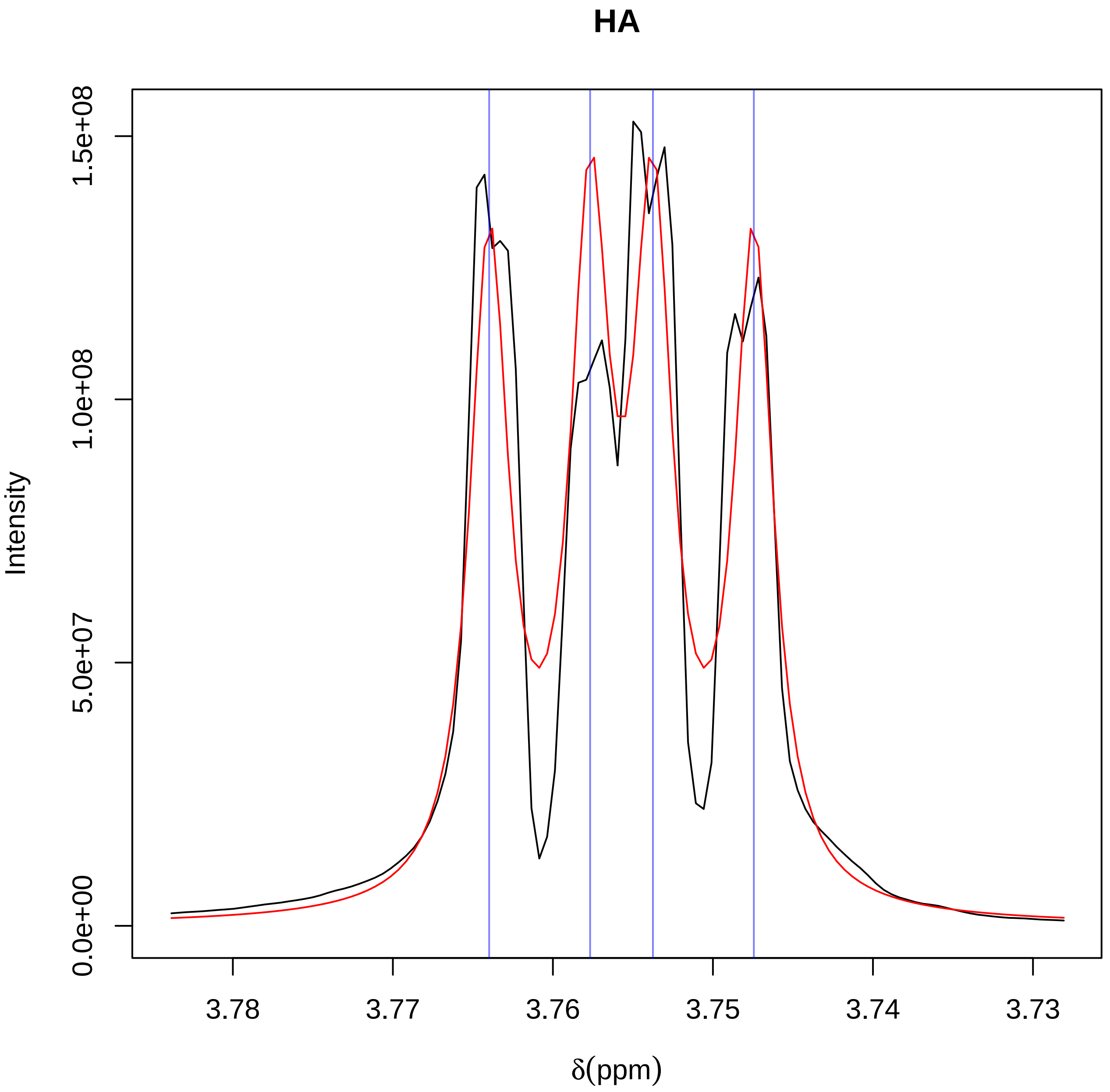

HA
Intensity
0.0e+00
5.0e+07
1.0e+08
1.5e+08
3.78
3.77
3.76
3.75
3.74
3.73
δ(ppm)

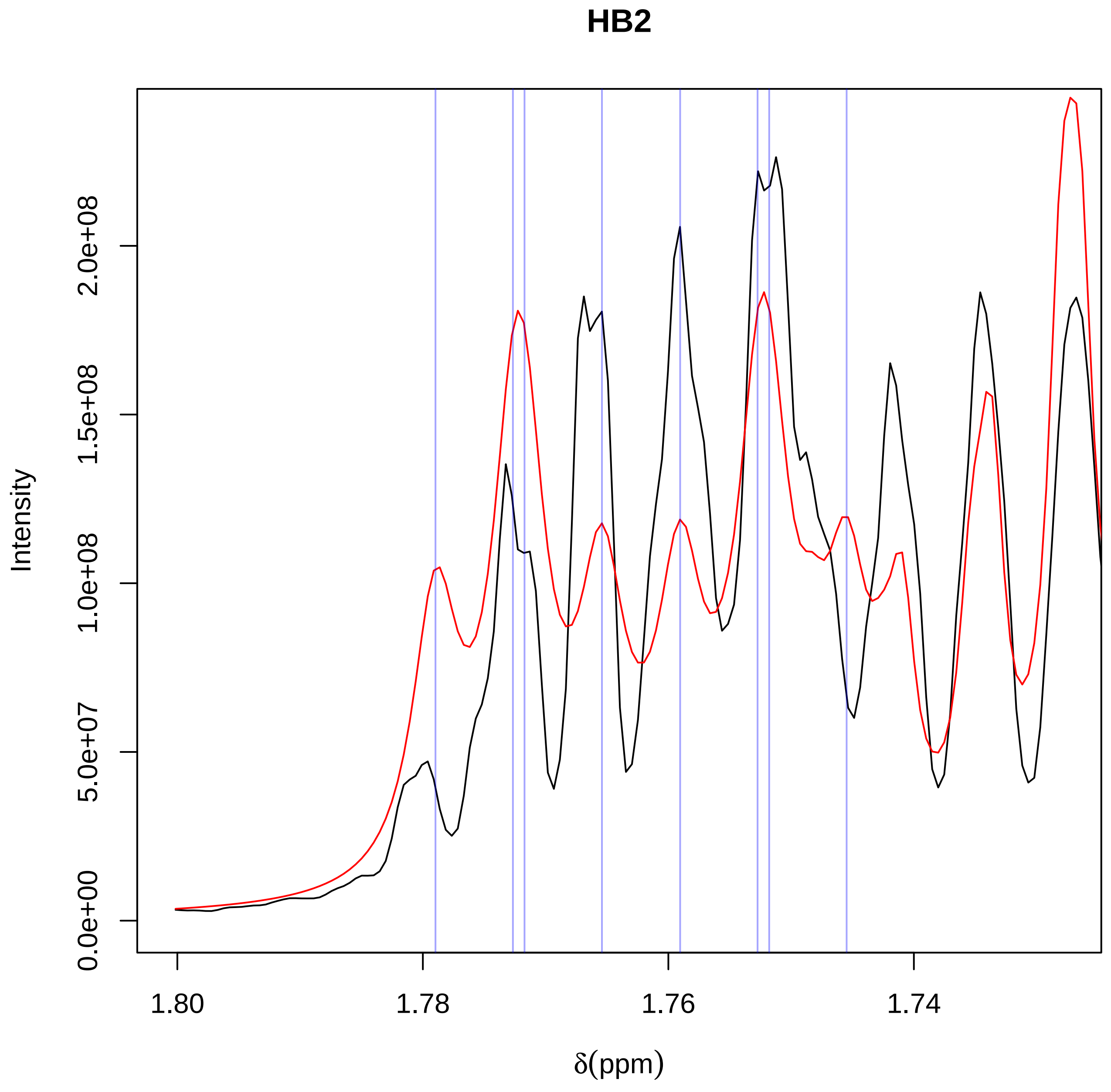
HB2
Intensity
0.0e+00
5.0e+07
1.0e+08
1.5e+08
2.0e+08
1.80
1.78
1.76
1.74
δ(ppm)

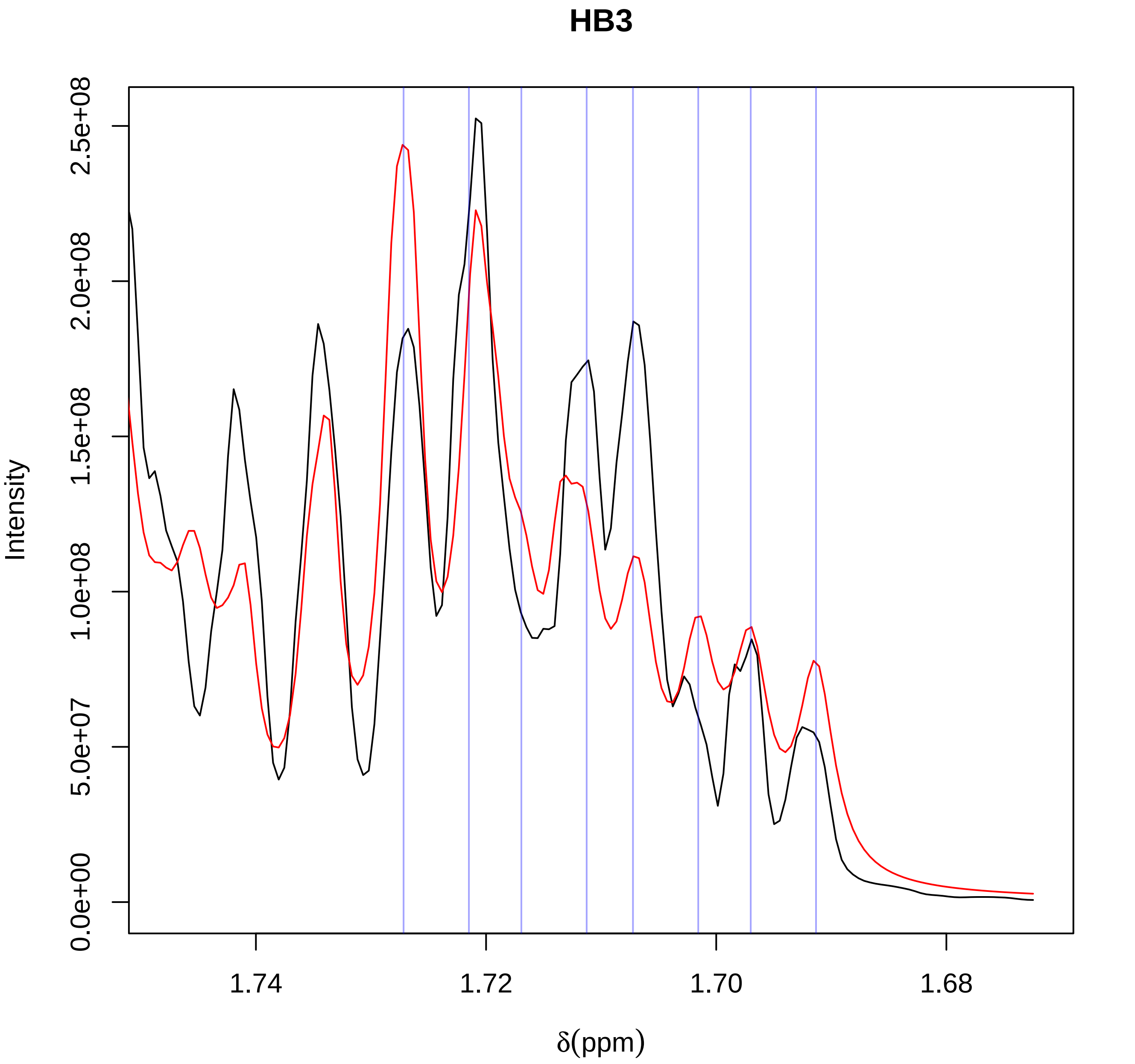
HB3
Intensity
0.0e+00
5.0e+07
1.0e+08
1.5e+08
2.0e+08
2.5e+08
1.74
1.72
1.70
1.68
δ(ppm)

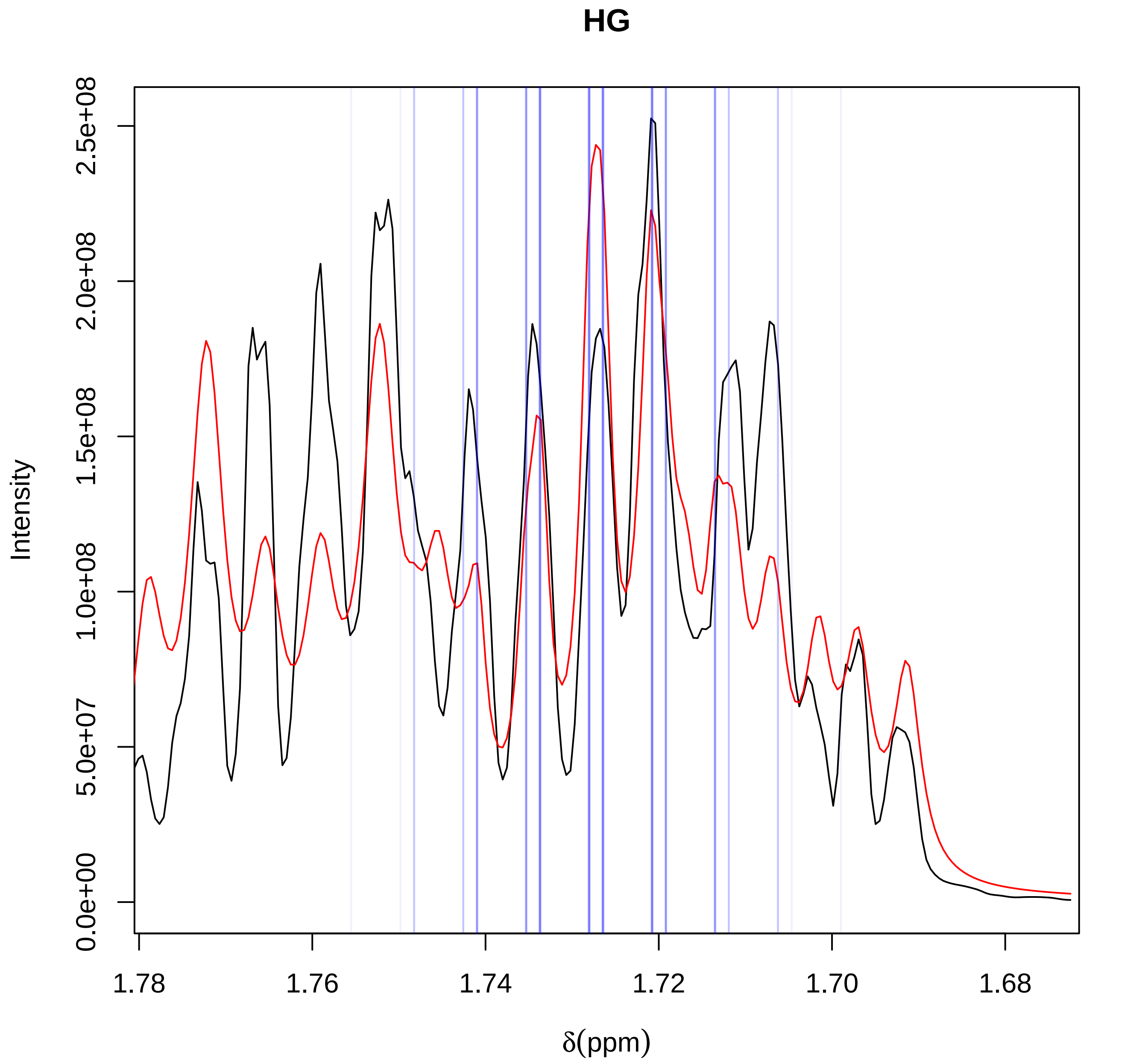

HG
Intensity
δ(ppm)
0.0e+00
5.0e+07
1.0e+08
1.5e+08
2.0e+08
2.5e+08
1.78
1.76
1.74
1.72
1.70
1.68

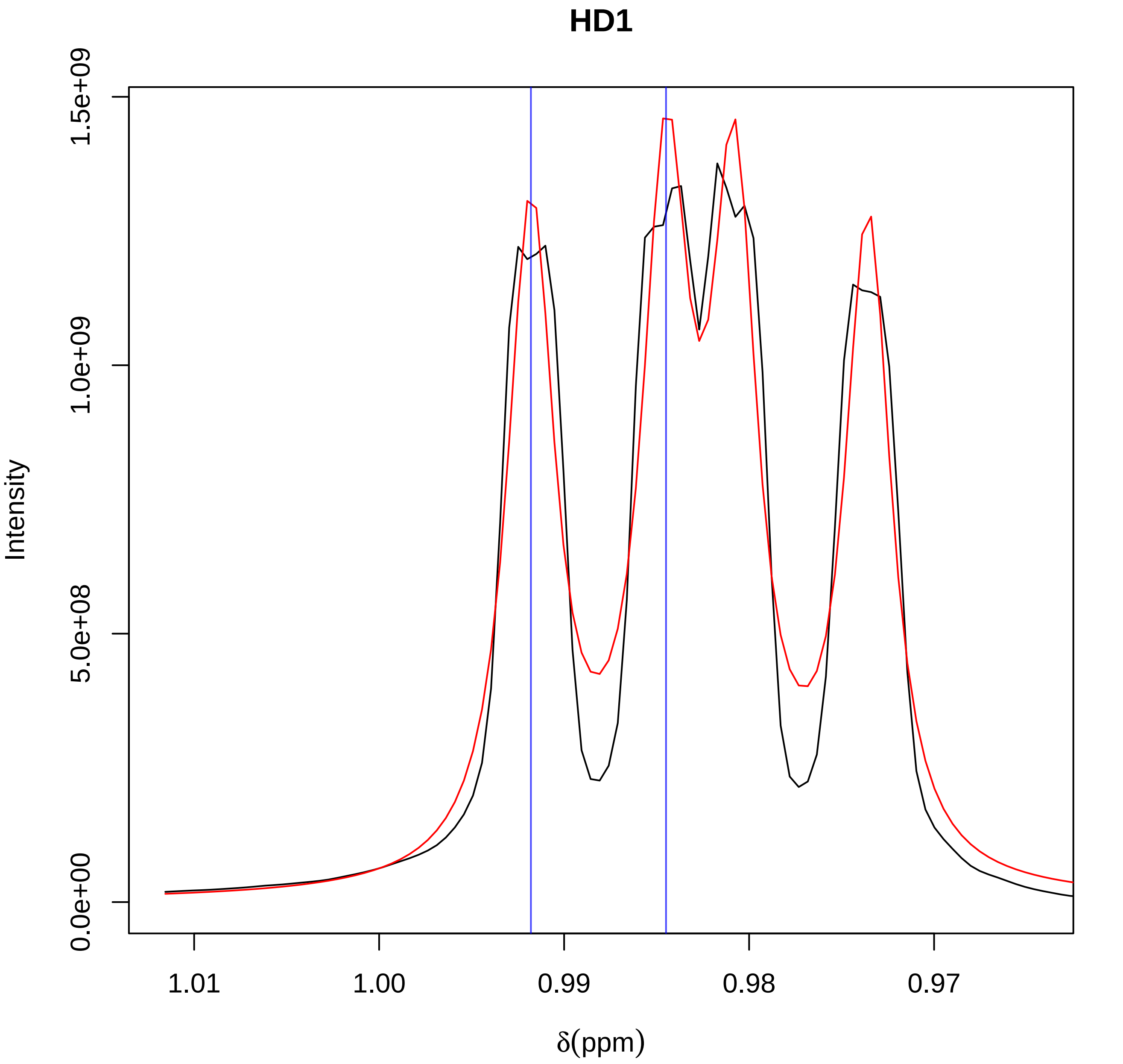
HD1
Intensity
0.0e+00
5.0e+08
1.0e+09
1.5e+09
1.01
1.00
0.99
0.98
0.97
δ(ppm)

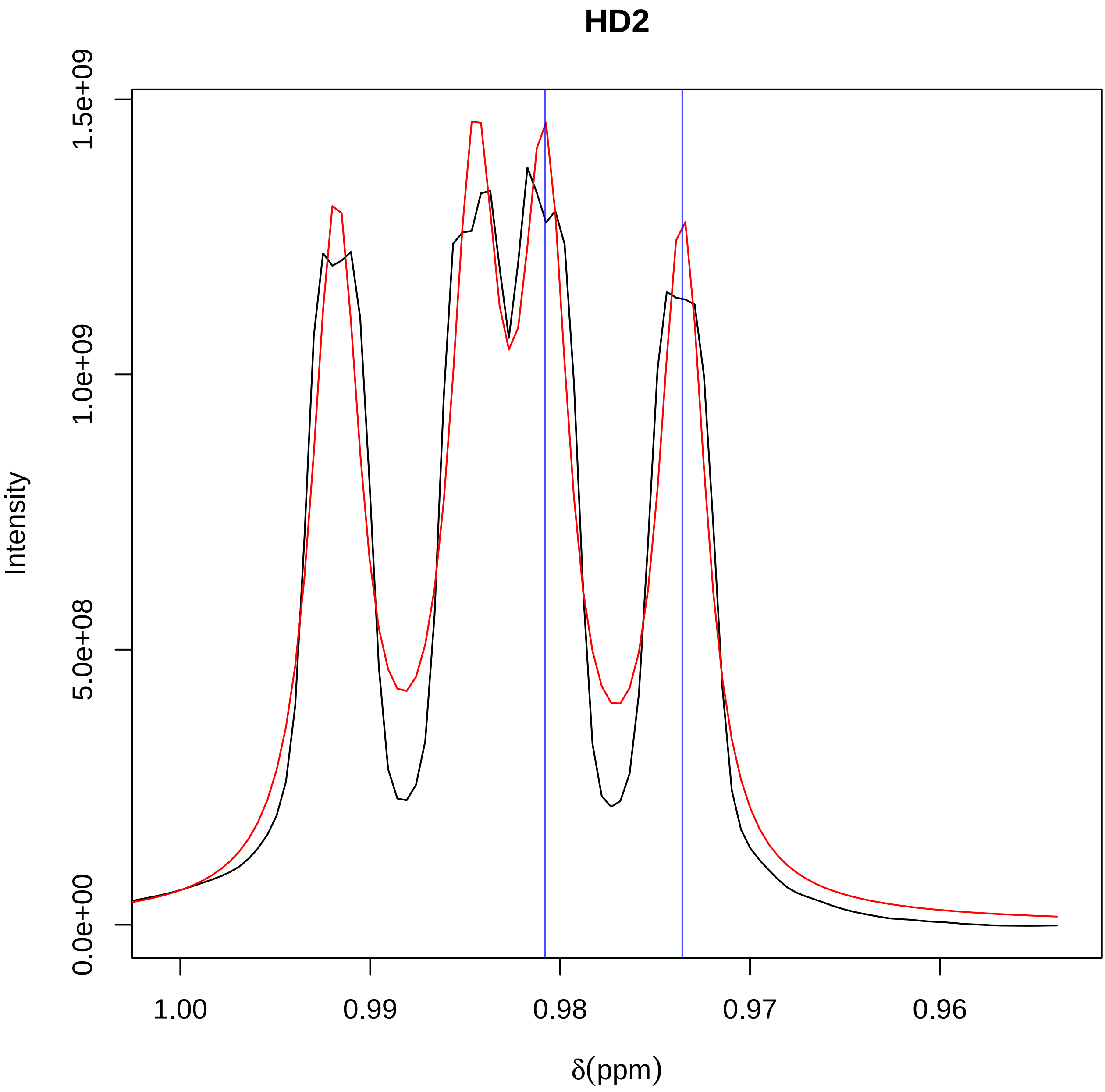

HD2
Intensity
0.0e+00
5.0e+08
1.0e+09
1.5e+09
1.00
0.99
0.98
0.97
0.96
δ(ppm)
